# Mortality Trends Related to Diabetes Mellitus and Heart Failure: A Retrospective Study

**DOI:** 10.7759/cureus.90376

**Published:** 2025-08-18

**Authors:** Bantu Prushni, Harnoor Dhillon, Dhwani Patel, Xinyu Lu, Maheswari Pulluru, Jeremy Edwards

**Affiliations:** 1 Internal Medicine, Sri Venkateswara Medical College, Tirupati, IND; 2 Internal Medicine, Bharati Vidyapeeth (Deemed to Be University) Medical College, Pune, Pune, IND; 3 Internal Medicine, Our Lady of Fatima University College of Medicine, Valenzuela, PHL; 4 Otorhinolaryngology, Shanghai Pudong Hospital, Shanghai, CHN; 5 Internal Medicine, Government Medical College, Srikakulam, Srikakulam, IND; 6 Internal Medicine, Eric Williams Medical Sciences Complex, Mt. Hope, TTO

**Keywords:** age-adjusted mortality rate, cdc mcd, diabetes mellitus, heart failure, retrospective study

## Abstract

Introduction: Diabetes mellitus (DM) is a major cause of mortality, and its association with heart failure (HF) remains underexplored. Understanding this relationship is essential to identifying high-risk populations and developing targeted public health interventions.

Aims: This study aims to analyze mortality trends and demographic disparities in DM with HF as a contributing cause.

Methods: A retrospective observational study was conducted using the Centers for Disease Control and Prevention (CDC) Multiple Causes of Death (MCD) database to assess mortality trends in individuals aged 25 years and older in the United States from 1999 to 2020. The study included deaths where DM (International Statistical Classification of Diseases and Related Health Problems, Tenth Revision (ICD-10): E10-14) was listed as the underlying cause and HF (ICD-10: I50) as a contributing cause. Data were analyzed by age, gender, race, geographic region, and place of death. Age-adjusted mortality rates (AAMR) and annual percent change (APC) were calculated.

Results: A total of 273611 deaths were recorded. The AAMR for DM with HF showed a small increase from 1999 to 2005 (APC: +0.31), followed by a sharp, significant decline from 2005 to 2010 (APC: -5.24), and started to rise steadily from 2010 to 2020 (APC: +1.88). The highest mortality was observed in females (n = 139882, 51.10%), White individuals (n = 225333, 82.40%), and in metropolitan areas (n = 209482, 76.50%). Temporal trends showed an increasing AAMR in males (+2.66 APC post-2010) and increasing AAMR amongst Asian or Pacific Islander individuals (+1.66 APC post-2010), who had the lowest overall mortality rates, indicating evolving disparities.

Conclusion: Overall, DM and HF-related mortality trends have shifted with disparities in gender, race, and location. Targeted interventions addressing comorbid management, preventive strategies, and social determinants of health are critical to mitigating excess mortality in high-risk populations concerning uptrends.

## Introduction

Diabetes mellitus (DM) encompasses a spectrum of metabolic disorders characterized by chronic hyperglycaemia resulting from defects in insulin secretion, action, or both [1]. Surveillance data from the Centers for Disease Control and Prevention (CDC) indicate that approximately 8.5% of U.S. adults were diagnosed with diabetes between 2017 and 2021, with no significant changes in prevalence during this period [2]. Diabetes remains a major contributor to mortality in the United States (U.S.), with diabetes-related mortality rates (DRMR) reaching 31.1 per 100,000 population in 2021 [2].

Heart failure (HF) is defined as a syndrome with symptoms and/or signs due to a structural or functional cardiac abnormality [3]. HF has emerged as a critical global health priority, currently affecting approximately 64 million individuals worldwide [4], reflecting the substantial disease burden. Epidemiological data demonstrate a bidirectional pathophysiological relationship between HF and DM, with each condition serving as an independent risk factor for the development of the other [5,6]. The Framingham study demonstrated that DM increases the incidence of HF by 2.4-5 fold [7], while 26-43% of HF patients exhibit comorbid DM [8,9]. The pathophysiological interplay between DM and HF stems from shared mechanistic pathways that collectively drive disease progression in both conditions [10]. Key contributing factors include chronic myocardial inflammation, oxidative stress, insulin resistance, neurohormonal dysregulation, endothelial dysfunction, progressive fibrotic remodelling, autonomic nervous system dysfunction, and metabolic disturbances leading to lipotoxic myocardial injury [11,12]. All of them contribute to the development of diabetes-associated cardiac dysfunction (diabetic cardiomyopathy). Additionally, social determinants of health - including socioeconomic status, access to healthcare, and health literacy - have been shown to independently influence cardiovascular outcomes and exacerbate both DM and HF, further compounding morbidity and mortality risk [11,12].

Furthermore, the coexistence of HF and DM portends significantly poorer outcomes and higher mortality rates compared to either condition in isolation. Clinical outcomes of HF are significantly worsened by comorbid DM, manifesting as elevated cardiovascular mortality, a higher rate of readmissions, and prolonged duration of hospitalization [13,14]. While prior studies have analyzed the impact of DM on mortality outcomes in HF patients, the reciprocal relationship, particularly the role of HF and its contribution to diabetes-associated mortality, remains inadequately investigated. The CDC Multiple Causes of Death (MCD) database provides a unique opportunity to examine how HF interacts with DM, offering a more comprehensive view of mortality patterns.

The aim of this study is to assess DM-associated mortality where HF is a contributing cause of death. This study uses the CDC MCD database, examines mortality trends from 1999 to 2020, then stratifies data by sex, race, and geographic location, and identifies disparities in mortality patterns.

## Materials and methods

The CDC Wide-Ranging Online Data for Epidemiologic Research (WONDER) MCD database was used for conducting the retrospective original research study [15]. Publicly accessible mortality data, including de-identified death certificate information for every death reported in the U.S., were used in the study. Data extraction was performed on April 15, 2025. Since the dataset includes de-identified information that is publicly accessible, the study was deemed to be non-human participant research, did not require consent, and was therefore exempt from institutional review board (IRB) approval.

The CDC WONDER MCD database was used to obtain mortality statistics for 1999-2020. Since DM-related mortality in younger demographics is uncommon, the study only recruited participants who were 25 years of age or older. In order to evaluate the co-occurrence of these illnesses, HF (I50) was chosen as the multiple cause of death and DM (E10-E14) as the underlying cause of death. To examine differences in mortality outcomes, demographic factors such as gender (male and female) and race/ethnicity (American Indian or Alaska Native individuals, Asian or Pacific Islander individuals, Black or African American individuals, White individuals) were included. Urbanization, according to the 2013 classification, which divided metropolitan cities into large central metro, large fringe metro, medium metro, and small metro, and non-metropolitan cities into micropolitan and non-core areas, was among the geographic variables. Furthermore, the location of death was classified as a medical facility that includes both inpatient and outpatient medical facilities, the descendant’s home, a hospice facility, or a nursing home. In order to enable accurate comparisons across time, mortality rates were standardized using age-adjusted rates per 1,000,000 population, with adjustments based on the U.S. Standard Population from 2000.

The demographic and geographic factors were summarized using descriptive statistics, such as absolute numbers and percentages. Those who do not meet the above variables were excluded from the study. For each of the subgroups, age-adjusted mortality rates (AAMRs) were computed using the CDC WONDER MCD database. In order to assess temporal trends, annual percent changes (APCs) in DM-related mortality with HF as a contributory cause were calculated using Join Point Regression Analysis (Join Point Software Version 5.3.0.0, November 2024; Statistical Research and Applications Branch, National Cancer Institute, Bethesda, MD, USA). In order to find statistically significant changes in mortality patterns across various demographic and geographic categories, trends were evaluated during the 1999-2020 research period.

## Results

In the year 1999-2020, the CDC MCD database recorded 55,317,623 deaths in the U.S. among individuals aged 25 years and older. Among these, deaths where DM (International Statistical Classification of Diseases and Related Health Problems, Tenth Revision (ICD-10): E10-E14) was listed as the underlying cause of death and HF (ICD-10: I50) was recorded as a multiple cause of death were included in the study (n = 273611, 0.49% of total deaths). The crude mortality rate for DM with HF as a contributing cause was 61.2 per 1,000,000 population. Deaths due to causes other than these criteria were excluded.

Among the total deaths analyzed, males accounted for 133729 (48.90%), while females accounted for 139882 (51.10%). The mortality rate for DM with HF as a contributing cause was higher in females compared to males, indicating a potential demographic disparity.

Regarding racial distribution, the highest proportion of deaths occurred amongst White individuals (n = 225333, 82.40%), followed by Black or African American individuals (n = 39094, 14.30%), Asian or Pacific Islander individuals (n = 6270, 2.30%), and American Indian or Alaska Native individuals (n = 2914, 1.10%). The mortality burden was highest among White people, highlighting racial disparities in mortality trends related to DM and HF.

A majority of deaths occurred in participants residing in metropolitan areas (n = 209482, 76.50%), while non-metropolitan areas accounted for 64129 (23.40%) deaths. Regarding the place of death, most deaths occurred in medical facilities (n = 103776, 38.00%), followed by decedents' homes (n = 84018, 30.70%), nursing homes or long-term care facilities (n = 69387, 25.40%), and hospice facilities (n = 7032, 2.60%).

Overall temporal trends

**Figure 1:**
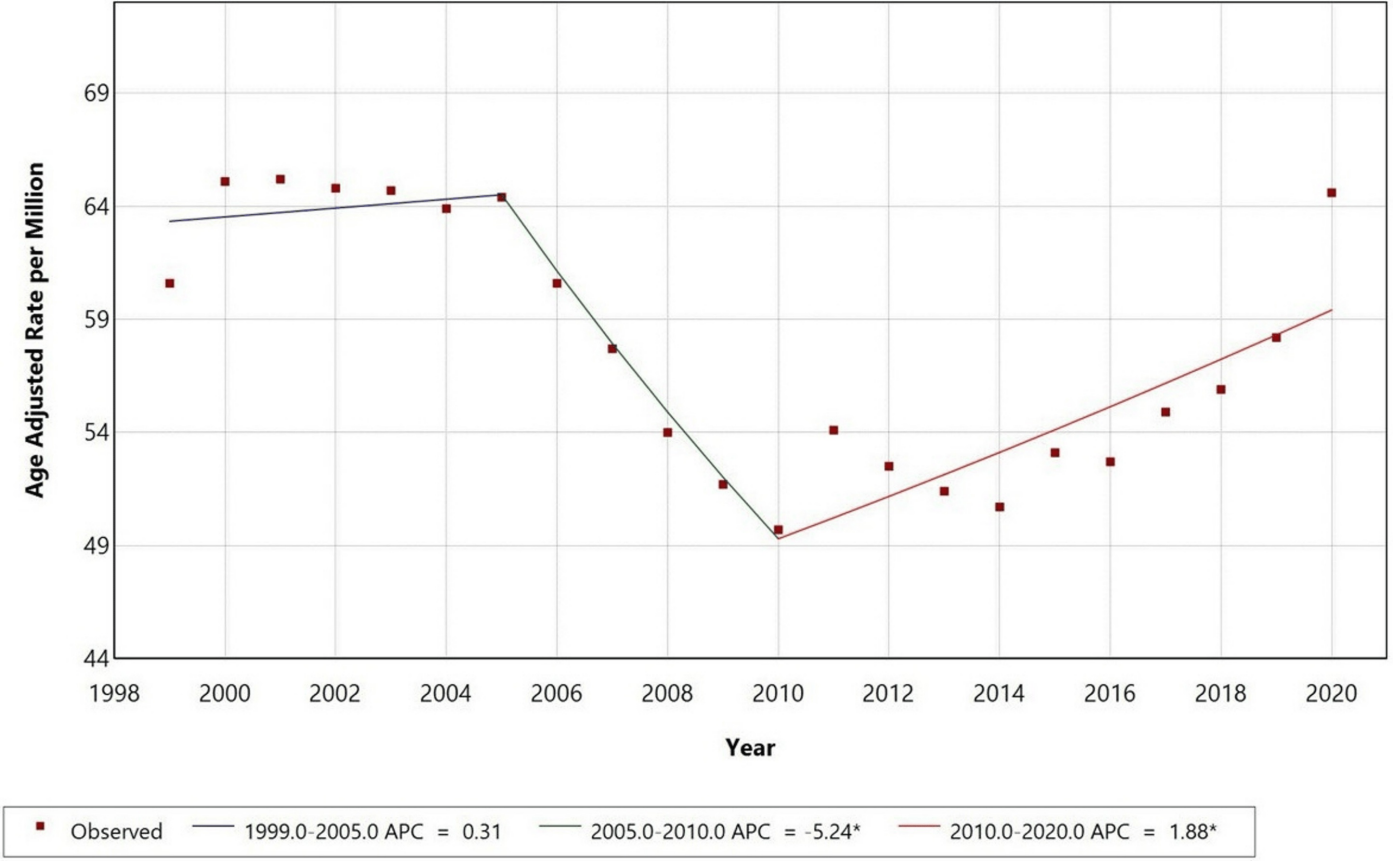
Overall age-adjusted mortality rates (AAMRs) among adults aged 25 years and older in the United States, 1999-2020 * indicates that the annual percent change (APC) is significantly different from zero at the alpha = 0.05 level. Final selected model: 3 joinpoints.

From 1999 to 2020, the AAMR for DM with HF as a contributing cause was almost stable from 1999 to 2005, after which it showed a decline till 2010, followed by a significant increase till 2020. The AAMR changed from 64 in 1999 to 64.5 in 2005, with an APC of +0.31; it then declined to 49.5 by 2010 with an APC of -5.24. Post this, the AAMR increased to 59 by 2020 with an APC of +1.88. Significant inflection points were observed in 2005 and 2010, indicating possible treatment advances, changes in disease prevalence, and lifestyle habits of the general public. Figure 1 presents AAMRs among adults aged 25 years and older in the U.S. from 1999 to 2020.

**Figure 2:**
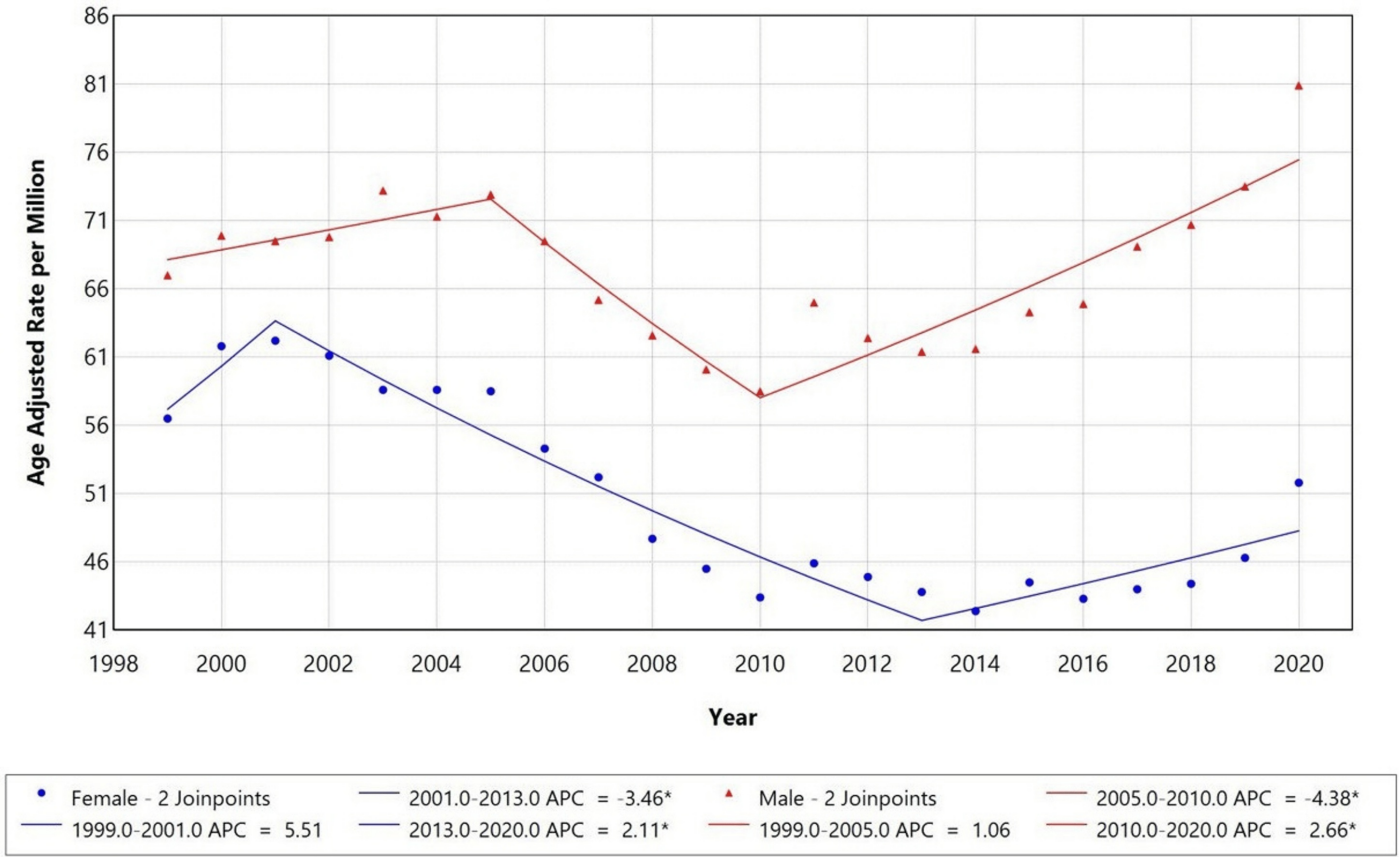
Trends in sex-stratified age-adjusted mortality rates (AAMRs) among adults aged 25 years and older in the United States, 1999-2020. * indicates that the annual percent change (APC) is significantly different from zero at the alpha = 0.05 level.

When stratified by gender, males had a higher AAMR (AAMR: 68) compared to females (AAMR: 57) in 1999. Both groups exhibited different trends, with APCs of +1.06 and +5.51 from 1999 to 2005 and 2001, respectively. Females showed a significant decrease in mortality from 2001 to 2013 (APC: -3.46), while the opposite trend was observed from 2013 to 2020 (APC: +2.11). Similarly, mortality in males declined from 2005 to 2010 (APC: -4.38), which then proceeded to rise from 2010 to 2020 (APC: +2.66). Figure 2 presents AAMRs among adults aged 25 years and older in the U.S. from 1999 to 2020, stratified by sex.

**Figure 3:**
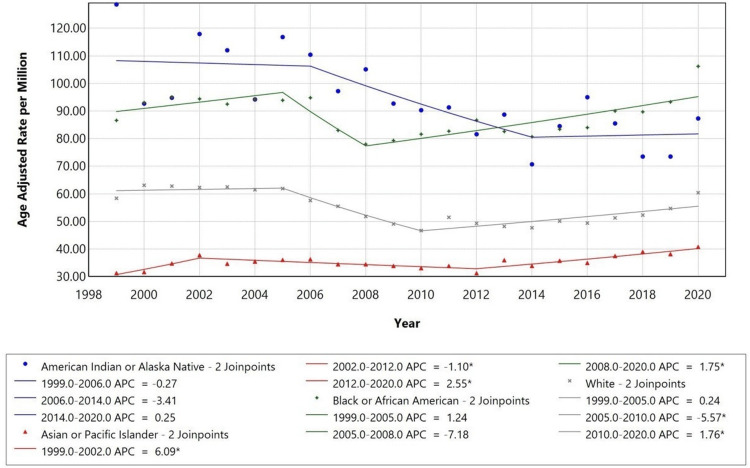
Trends in age-adjusted mortality rates (AAMRs) stratified by race among adults aged 25 years and older in the United States, 1999-2020. * indicates that the annual percent change (APC) is significantly different from zero at the alpha = 0.05 level.

Racial disparities were observed in mortality trends. In 1999, American Indian or Alaska Native individuals had the highest AAMR (AAMR: 109), followed by Black or African American individuals (AAMR: 90), White individuals (AAMR: 60), and Asian or Pacific Islander individuals (AAMR: 30). Over the course of the years being studied, each racial group showed varied inflection points. Amongst the American Indian or Alaska Native individuals, the AAMR decreased up till 2014 (APC: -3.41) and was further stable till 2020. The APC for White individuals from 1999 to 2005 was +0.24, indicating almost stable mortality rates. There was a decrease in mortality from 2005 to 2010 (APC:-5.57), then showing a rise up till 2020 (APC: +1.76). On the other hand, AAMR in Black or African American individuals showed a significant decrease from 2005 to 2008 (APC of -7.18), with a slight increase till 2020 (APC: +1.75). Finally, amongst Asian or Pacific Islander individuals, there was a significant rise from 1999 to 2002 (APC: +6.09) and another rise from 2012 to 2020 (APC: +2.55). Figure 3 presents AAMRs among adults aged 25 years and older in the U.S. from 1999 to 2020, stratified by race.

## Discussion

The CDC WONDER MCD database was used for conducting this retrospective original research study [15]. The data were used to assess the mortality trends in relation to DM (E10-E14) with HF (I50) as the contributing cause in the U.S. from 1999 to 2020 among adults aged 25 years and above. The overall crude mortality rate was 50.6 per million with a total of 273,651 deaths recorded during this period. Age-adjusted mortality trends for DM with HF were initially flat with a slight increase from 1999 to 2005 (APC: +0.31), followed by a sharp, significant decline from 2005 to 2010 (APC: -5.24), and started to rise significantly and steadily from 2010 to 2020 (APC: +1.88). The highest mortality was observed in females (51.10%), White individuals (82.40%), and in metropolitan areas (76.50%).

People with DM are over twice as likely to develop HF and also face worse hospital outcomes and prognosis. Despite managing blood sugar, the risk remains the same [16,17]. The precise biological connection between DM and HF is not completely understood. However, factors such as persistently high blood sugar, resistance to insulin, and elevated insulin levels play significant roles in promoting disease progression. DM can lead to heart damage both indirectly by affecting small and large blood vessels (micro and macrovasculopathy) and directly, by negatively impacting the myocardial tissue [18]. DM leads to structural alterations in the heart, including left ventricular hypertrophy and fibrosis, contributing to HF development [19]. A study showed that left ventricular diastolic dysfunction and HF with preserved ejection fraction are most common in type 2 DM patients [20].

Our study found an overall AAMR of 57.7 per million for DM with HF, with an APC of approximately +0.24% per year. An extended review of national health data showed that while deaths due to stroke and heart attacks declined in people with diabetes, mortality specifically due to HF did not improve [21]. A Danish study showed a decline in the incidence of HF in type 2 DM patients; the difference is likely due to improved treatment and differing inclusion criteria [22]. Another study showed increasing mortality in DM patients with HF, which is consistent with our findings of increasing AAMR [23].

This study found that initially, AAMR declined for both sexes post-2005, likely due to improved disease management. But post-2010, there is a significant increase in mortality in both sexes; previous research showed men are at higher risk of death due to DM and HF [23], which is consistent with our study. A study showed that females with HF experience less myocyte death and have a slower disease course [24], and estrogen may contribute to the improved outcomes seen in females [25]. Racial disparities were also observed, with American Indian/Alaska Native individuals and Black individuals experiencing the highest AAMR increase, which is consistent with prior studies [23,26], most likely due to higher prevalence, structural racism, socioeconomic status, and healthcare accessibility.

A notable disparity in mortality was identified between metropolitan (82.8%) and non-metropolitan (17.2%) areas. Previous research suggests that urban regions may benefit from better access to specialised cardiology and receive implantable cardioverter devices for HF management, potentially contributing to improved patient outcomes [27]. Even within metropolitan regions, health disparities continue to exist, often driven by socioeconomic inequalities. Evidence from a population-based study in China showed that individuals with lower levels of education, limited occupational opportunities, and reduced household wealth faced a greater risk of cardiovascular disease and higher mortality rates [28]. The elevated AAMRs observed in non-metropolitan regions are consistent with evidence showing that rural populations often face challenges such as limited healthcare resources, lower screening rates, and restricted access to advanced cardiovascular treatments.

Our study showed that overall AAMR was initially flat with a slight increase from 1999 to 2005 (APC: +0.31), followed by a sharp, significant decline from 2005 to 2010(APC: -5.24), and started to rise significantly and steadily from 2010 to 2020 (APC: +1.88). The initial decline is most likely due to advancements in medical and surgical interventions. And post-2010 rise is likely due to the ageing population in developed countries, COVID-19, which strained healthcare resources, and DM with HF patients are more vulnerable to COVID-19. As there is an increase in disease burden due to ageing populations, higher rates of hospitalisation and higher documentation of deaths are noted [24]. Gender-based analysis revealed that mortality rates among males consistently exceeded those of females throughout the study period. However, after 2013, a notable rise in female mortality was observed.

The rising mortality in DM-related HF highlights the need for further research in biological mechanisms, early detection, pharmacologic therapies, and public health needs. Targeted interventions are essential for high-risk groups, including minorities and rural populations. Integrated diabetes-cardiovascular care, improved healthcare access, and attention to social determinants like income and education are crucial. Sex-specific strategies and preventive efforts, such as promoting healthy lifestyles, are needed. Enhanced surveillance and policy reform can reduce disparities and support better outcomes, especially amid challenges like aging populations and post-COVID healthcare strain.

Limitations

This study has several limitations due to potential coding inaccuracies in death certificate data and the absence of information regarding comorbidities and treatment history in patients with DM and HF. Variations in diagnostic criteria and reporting practices over time may have influenced the observed trends. Being a retrospective observational study, it cannot establish causality. Additionally, factors such as socioeconomic status, healthcare access, and lifestyle behaviours were not included in the MCD database, potentially affecting the results. These unaccounted variables may influence the interpretation and generalizability of the findings in patients with DM and HF.

## Conclusions

This study uniquely examines two decades of national mortality data to assess trends in DM-associated deaths with HF as a contributing cause, revealing important demographic and geographic disparities. The rising post-2010 mortality, particularly among males and Asian or Pacific Islander individuals, underscores evolving epidemiological patterns. Findings emphasize the urgent need for targeted interventions - integrating diabetes and cardiovascular care, expanding preventive services, and addressing social determinants such as income, education, and healthcare access. Recognizing these disparities enables policymakers and clinicians to prioritize high-risk groups and tailor community-based strategies. While the study’s scope is limited by the retrospective design and reliance on death certificate data, its population-level insights fill a knowledge gap on the reciprocal mortality burden of DM and HF. Future work should incorporate clinical variables and prospective analyses to refine understanding and guide interventions aimed at reversing these unfavourable trends.
